# Is Fibromyalgia a Fashionable Diagnosis or a Medical Mystery?

**DOI:** 10.7759/cureus.44852

**Published:** 2023-09-07

**Authors:** Giustino Varrassi, Martina Rekatsina, Serge Perrot, Elyse Bouajina, Antonella Paladini, Stefano Coaccioli, Marco Antonio Narvaez Tamayo, Piercarlo Sarzi Puttini

**Affiliations:** 1 Pain Medicine, Paolo Procacci Foundation, Rome, ITA; 2 Pain Management, Basildon University Hospital, London, GBR; 3 Pain, Cochin University Hospital, Paris, FRA; 4 Rheumatology, Farhat Hached University Hospital Center, Sousse, TUN; 5 Life, Health and Environmental Sciences (MESVA), University of L'Aquila, L'Aquila, ITA; 6 Internal Medicine, Universita di Perugia, Terni, ITA; 7 Pain Medicine, Hospital Obrero, La Paz, BOL; 8 Rheumatology, Luigi Sacco University Hospital, Milan, ITA

**Keywords:** fibromyalgia diagnosis, pain diagnosis, chronic pain, pain, fibromyalgia, fibromyalgia criteria

## Abstract

Despite its prevalence, there is no clear-cut diagnostic path or treatment paradigm for fibromyalgia; this can lead to a multiplicity of symptoms and comorbid conditions that complicate care. “Overlapping symptoms” describe conditions that can occur concomitantly with fibromyalgia and include migraine, irritable bowel syndrome, obesity, and pelvic pain syndromes. A variety of pharmacologic and nonpharmacologic treatments are available for fibromyalgia, but treatment is best personalized for an individual and recognizes potential comorbidities. Opioids are not the recommended front-line treatment, cannabinoids hold promise but with limitations and nonpharmacologic options, such as aerobic or resistance exercise and cognitive behavior therapy, can play a very important but often underestimated role. Amitriptyline appears to be safe and effective in treating six of the main fibromyalgia domains: pain, disturbed sleep, fatigue, affective symptoms, functional limitations, and impaired cognition (“fibro fog”). Very low-dose naltrexone (2.5-4.5 mg) may offer analgesic and anti-inflammatory benefits to fibromyalgia patients, but further studies are needed. Fibromyalgia can be a devastating and debilitating condition for patients, and clinicians are challenged with its diagnosis and treatment as well. Further research as well as compassionate approaches to offering personalized care to those with fibromyalgia are required.

## Introduction and background

Despite its global prevalence, fibromyalgia is associated with protracted diagnostic delays, on average 2.3 years [1]. Patients often experience a confounding multiplicity of symptoms, including fatigue, chronic pain, insomnia, headache, gastrointestinal problems, and others [2]. In a survey of 800 fibromyalgia patients (with 84% females), 25% reported symptoms so debilitating that they could no longer work [1]. The characteristic classic symptoms of fibromyalgia are widespread tenderness and pain accompanied by fatigue, cognitive dysfunction (“fibro fog”), and anxiety and/or depression [3]. In real-world clinical practice, fibromyalgia patients often have a host of other symptoms and these symptoms can wax and wane unpredictably.

The etiology of fibromyalgia is not elucidated, but this condition may be part of a larger metabolic deficiency in the serotoninergic system [4]. Lacking diagnostic markers, this “invisible disease” has been labeled neurasthenia, fibrositis, fibromyositis, psychogenic rheumatism, and finally fibromyalgia [5-7]. Since fibromyalgia is a disease defined entirely by symptoms, doubts remain even to this day as to whether or not it is a real clinical condition [8]. Some of the most important evidence arguing in favor of its identity as a disease were brain scans showing increased neuronal excitation and amplification of pain signals [9,10]. A current proposition holds that fibromyalgia may be a spectrum with peripheral pain at one end and centralized pain at the other [9].

This article is a review of materials presented at the “Past, Present and Future in Pain Medicine, Second Edition” congress held in Tunis, Tunisia, from May 11 to 13, 2023. Materials were presented by the authors based on their research and clinical experience. The aim of this review is to describe the findings of this panel of experts on fibromyalgia.

## Review

Pain signaling in fibromyalgia

The neurotransmitters associated with pain signaling include glutamate, substance P, nerve growth factor (NGF), and serotonin (5HT-2A and 5HT-3A). The neurotransmitters that inhibit the transmission of pain signals are norepinephrine-5 serotonine (5HT-1A, 5HT-1B), dopamine, endogenous opioids, endocannabinoids, and gamma-aminobutyric acid (GABA) [3,11]. A patient with fibromyalgia has an imbalance with more pain-facilitating neurotransmitters, such as glutamate and substance P, and less pain-inhibiting neurotransmitters, such as GABA and norepinephrine. Pharmacologic treatments of fibromyalgia are predicated on the foundation of restoring a more balanced brain chemistry, but drug therapy is not always effective or may only be effective as part of a broader, more multifaceted treatment approach. This is particularly true when fibromyalgia patients present with symptoms other than the conventionally expected ones.

Comorbidities and overlapping symptoms

Compared to the general population, a higher proportion of fibromyalgia patients experience psychiatric and rheumatologic conditions, and these comorbidities can obscure diagnoses and complicate care [12]. Among these comorbidities are depressive disorder, anxiety disorder, bipolar disorder, rheumatoid arthritis, osteoarthritis, gout, vasculitis, coronary heart disease, hypertension, diabetes, irritable bowel syndrome, Crohn’s disease, cancer, peripheral neuropathy, and others [12]. Despite its importance in fibromyalgia care, the concept of a cluster of potential comorbidities is not well recognized in medicine or public health [13]. Fibromyalgia remains primarily a chronic pain condition, but many of its associated comorbidities are not [13]. When comorbid fibromyalgia is not properly diagnosed, clinicians and patients may erroneously assume the primary disease is poorly managed.

It is far more accurate to talk about “fibromyalgia syndrome” that includes so-called “overlapping symptoms” of multiple conditions. A new nosology has been proposed with the recognition of central sensitivity syndrome in fibromyalgia [14]. This is thought to be a plausible explanation for the frequently observed and comorbid relationship between irritable bowel syndrome and fibromyalgia [15]. Women with irritable bowel syndrome have an 8% to 41% prevalence of fibromyalgia, which may further overlap with chronic pelvic pain. Women with chronic pelvic pain have a 4% to 31% prevalence of fibromyalgia [16]. Interstitial cystitis or bladder pain syndrome, lacking a clear etiology and no definitive treatment, has been associated with both fibromyalgia and irritable bowel syndrome, along with a variety of other conditions including anxiety and depression [17]. A survey of 205 interstitial cystitis patients compared to 117 controls found that 17.7% of interstitial cystitis patients reported fibromyalgia symptoms compared to 2.6% of controls [18].

Our understanding of these fibromyalgia comorbidities remains limited. For example, obesity has been recognized as a common fibromyalgia comorbidity that exacerbates symptoms. In a study of 215 fibromyalgia patients, obesity in fibromyalgia patients was related to higher sensitivity to pain, reduced physical strength, less sleep, and increased restlessness during sleep compared to controls [19]. The overlapping comorbidities can expand further, since type 2 diabetes mellitus (T2DM) is likewise comorbid with both fibromyalgia and obesity [20].

Among patients with rheumatoid arthritis, axial spondylarthritis, and psoriatic arthritis, the incidence of fibromyalgia is higher than in the general population [21]. These comorbid conditions can adversely impact treatments and outcomes. It can also be challenging to distinguish pain symptoms attributable to a rheumatic condition versus centralized fibromyalgia pain.

In fibromyalgia patients, the symptoms of comorbid conditions can be more severe than symptoms that occur in isolation. For instance, patients with fibromyalgia and comorbid migraine have more intense headaches, a greater likelihood of severe headaches, and more symptoms of depression compared to those with migraine alone [22]. About 20% to 36% of migraineurs have fibromyalgia [23-25]. The relationship between fibromyalgia and migraines appears to be bidirectional, so those with one condition are statistically more likely to have the other [25].

Since fibromyalgia patients are at an elevated risk for comorbid conditions, such as psychiatric disorders and rheumatic conditions, it is important to screen fibromyalgia patients for such conditions rather than assuming that somatic symptoms are all attributable to fibromyalgia. Identifying comorbidities can delineate effective treatment strategies, improve outcomes, and may even reduce symptoms of fibromyalgia itself [12].

Differential diagnosis is complicated by the fact that fibromyalgia mimics the symptoms of other conditions, such as lupus, multiple sclerosis, rheumatoid arthritis, polymyalgia rheumatica, axial spondyloarthritis, thyroid disease, T2DM, anemia, and chronic fatigue syndrome [26]. Patient history can be helpful as early-life trauma, infections, and physical or emotional stress can be associated with fibromyalgia as well as chronic fatigue syndrome. The diagnostic process may be further hampered by the fact that not all nations recognize fibromyalgia and, even where fibromyalgia is an accepted diagnosis, many clinicians are dubious about it [27,28].

The pathway to care typically starts with a first-level clinician, such as a general practitioner, with a subsequent referral to a rheumatologist or other specialist [27]. The main differential diagnoses are statin-induced myalgias, hypothyroidism, various inflammatory or rheumatic conditions, neuropathic conditions, sleep apnea syndrome, somatoform disorders such as anxiety or depression, and viral illnesses [26,27]. Red flags include fever, joint swelling or tenderness, joint warmth, photosensitivity, alopecia, rash, abnormal values in a lab test, neurological signs and symptoms, and pain that does not respond to conventional pharmacological analgesia [29]. Classic symptoms of fibromyalgia include widespread pain, fatigue, and disturbed sleep, but fibromyalgia can also reduce function, depress mood, and impair cognition [29].

Treating fibromyalgia

Fibromyalgia is usually treated with a multipronged approach that includes patient education, nonpharmacological strategies such as exercise or cognitive behavioral therapy, and pharmacological treatments such as gabapentinoids, selective serotonin reuptake inhibitors (SSRIs), serotonin-norepinephrine reuptake inhibitors, tricyclic antidepressants, low-dose naltrexone, nonsteroidal anti-inflammatory drugs (NSAIDs), opioids, and cannabinoids (Table 1) [27]. A recent study in 65 fibromyalgia patients treated with a novel form of ozone therapy found >50% symptomatic relief in 70% of the patients with no side effects reported [30]. Ozone therapy is being used to treat a number of chronic conditions and appears to exert a mild and transient state of controlled oxidative stress that upregulates the antioxidant system and modulates the immune system [30]. Because it has very few side effects, it is recommended for treating fibromyalgia patients who have not responded to other therapies [30].

**Table 1:** Summary of proposed therapies for fibromyalgic patients

Pharmacological	Nonpharmacological
Gabapentin, pregabalin	Exercise
Antidepressants	Cognitive behavioral therapy (CBT)
Opioids	Yoga
Low-dose naltrexone	T’ai Chi
Nonsteroidal anti-inflammatory drugs (NSAIDs)	Qi Gong
Cannabinoids	Acupuncture
Nutrition	Hydrotherapy
	Cupping
	Traditional Chinese medicine
	Mindfulness
	Trans-cranial stimulation

Advances in fibromyalgia studies caused the European League Against Rheumatism (EULAR) to issue the 2017 recommendations that shift away from reliance on expert opinion to more evidence-based recommendations [31,32]. Despite a limited number of studies, low-dose amitriptyline, duloxetine or milnacipran, tramadol, pregabalin, and cyclobenzaprine are supported by high-quality evidence (Table 2) [31].

**Table 2:** Summary of interesting pharmacological treatments suggested in guidelines NSAID, nonsteroidal anti-inflammatory drug; SSRI, selective serotonin reuptake inhibitor Source: Ref. [31]

Drug	Dosages	Results	Comments
Amitriptyline	10-50 mg/day, 8-24 weeks	Low	No differences with placebo reported in the literature
Pregabalin	600 mg/day	High	Increased likelihood of withdrawal because of adverse events
NSAIDs	Several	Low	Adverse events are more than the advantages
Duloxetine	20-120 mg/day, 12-28 weeks	Moderate	Dropout because of side effects significantly higher than with placebo
Milnacipran	100-200 mg/day, 12-27 weeks	High	Dropout rate is double compared to placebo, but not serious adverse events
SSRIs	Several	Moderate to high	Acceptability and tolerability similar to placebo
Tramadol	150 mg/day, combined with paracetamol 1300 mg/day	High	No significant discontinuation rate, compared to placebo, due to adverse events

Gabapentin and Pregablin

The gabapentinoids, pregabalin and gabapentin, are anticonvulsants that act by reducing the calcium-dependent release of glutamate, norepinephrine, calcitonin gene-related peptide, and substance P. As such, they treat neuropathic pain as well but with some well-known limitations. Their analgesic benefit may be modest; also, there are potentially treatment-limiting side effects and not all patients respond to them [33]. There is mixed evidence in the literature about the effectiveness of gabapentinoids against painful symptoms of fibromyalgia [34,35]. The most frequently reported side effects with pregabalin and gabapentin are dizziness, somnolence, headache, and edema [33]. Overall, gabapentin and pregabalin offer greater pain control for fibromyalgia patients than placebo, but the response appears to be somewhat dose dependent, and they are not durable since about a third of patients reportedly lose response at around six months [33]. An important gap in our understanding of fibromyalgia treatments is the absence of head-to-head studies of various agents in this population.

Amitriptyline and Other Antidepressants

Amitriptyline is a tricyclic antidepressant that is often recommended as monotherapy or part of a combination regimen to treat fibromyalgia pain. One of the first tricyclic antidepressants to be approved for the market, amitriptyline inhibits serotonin and norepinephrine reuptake and may be active at histaminergic, muscarinic, and norepinephrine receptors [36]. Amitriptyline is effective against six of the main domains of fibromyalgia: pain, sleep disturbances, fatigue, affective symptoms, functional deficits, and cognitive impairment [37]. Although there is a paucity of studies, amitriptyline at low doses in the range of 10-75 mg/day is considered effective in managing fibromyalgia pain [38]. Concerns regarding amitriptyline in this population are tachyphylaxis and cardiotoxic effects [36,37].

Serotonin and norepinephrine reuptake inhibitors have been shown to be effective in decreasing pain intensity in fibromyalgia patients. Duloxetine is described as a balanced serotonin and noradrenaline reuptake inhibitor and is indicated to treat diabetic peripheral neuropathy, major depressive disorder, and urinary stress incontinence [39]. It has been used to manage fibromyalgia pain both alone and in combination with other drugs. The typical dose range is 60-120 mg/day. The Food and Drug Administration (FDA) has issued a black box warning about suicidality associated with duloxetine, and its other potentially serious side effects include tachycardia and hypertension [40].

Milnacipran, at doses of 100-200 mg/day, is a serotonin-norepinephrine reuptake inhibitor and is one of only a handful of agents expressly developed to treat fibromyalgia [41]. While it reduces pain and fatigue, its benefits may be modest and side effects include sleep-related disturbances and depression [42,43].

There are a few head-to-head studies on fibromyalgia agents. A meta-analysis of various fibromyalgia treatments indirectly compared pregabalin, amitriptyline, and duloxetine at various dosages and reported that amitriptyline 25 mg had superior results, followed next by pregabalin 450 mg [44].

Opioids

A study, including 14 fibromyalgia, 10 chronic low back pain, and 6 healthy control patients who received lumbar puncture, was conducted to analyze the amount of Met-enkephalin-Arg6-Phe7 (MEAP) and nociceptin immunoreactive materials in their cerebrospinal fluid. The fibromyalgia patients had higher MEAP concentrations in their cerebrospinal fluid, more systemic symptoms, and lower pain thresholds compared to the back pain patients and the control group [45]. Thus, endogenous opioids may be deployed to help manage fibromyalgia pain, but the question arises as to the role of exogenous opioid therapy. Evidence of the effectiveness of opioid therapy against fibromyalgia pain is limited with no evidence advocating long-term opioid treatment [46]. For that reason, leading pain specialty societies advise that opioids should be avoided for treating fibromyalgia [32,47]. Prolonged exposure to opioids can cause tolerance, provoking distressing withdrawal symptoms when the drug is necessarily discontinued or dosage reduced; there is also the potential risk of opioid use disorder [48]. Despite these known risks, opioids are frequently prescribed to this population and are associated with a greater burden of illness [49].

Tramadol, considered a weak opioid agonist, is the exception to the rule. Tramadol has a mechanism of action related to serotonin reuptake inhibition. In a 91-day study of fibromyalgia patients taking a fixed-dose combination product of 37.5 mg tramadol and 325 mg of paracetamol (acetaminophen), the tramadol-paracetamol patients had significantly less pain and better function than control patients and no serious adverse events were reported [50].

Low-Dose Naltrexone

At doses of 50-100 mg, naltrexone is approved in the United States to treat opioid or alcohol use disorder and opioid dependence, but at very low doses of 2.5-4.5 mg, the agent has analgesic and anti-inflammatory properties, confirmed in a study of 31 fibromyalgia patients [51]. In a study of eight fibromyalgia patients, it was found that low-dose naltrexone over 10 weeks reduced fibromyalgia pain by 15%, decreased overall fibromyalgia symptoms by 18%, and limited the presence of pro-inflammatory cytokines as well [52]. In a systematic review of studies of low-dose naltrexone in fibromyalgia patients, the regimen was found to be effective in controlling fibromyalgia symptoms with no severe adverse events reported [53]. This off-label use of low-dose naltrexone is not widely prescribed or well known among clinicians.

Nonsteroidal Anti-inflammatory Drugs

NSAIDs are typically not effective in treating fibromyalgia pain because they have peripheral rather than central mechanisms of action [54-56]. Since many are available over-the-counter, they may be taken by patients trying to seek pain relief, but are generally not effective.

Cannabinoids

Despite legalization efforts and a wealth of new research, clinicians are still not confident about how to prescribe cannabinoids, what forms of cannabinoids and routes of administration to recommend, or how well cannabinoids will work for fibromyalgia symptoms [57]. Cannabinoid receptors, known as CB1 and CB2, are part of the body’s endocannabinoid system. CB1 receptors are mostly centrally located and mediate euphoric and analgesic effects. CB1 can also reduce inflammation and blood pressure. CB2 receptors, on the other hand, are mainly located in the periphery and have immunomodulatory and anti-inflammatory effects [58]. Brain-mapping studies have suggested that tetrahydrocannabinol (THC), the active and psychoactive component of cannabis, does not selectively impact the limbic regions; instead, it works by interfering with sensory processing, which, in turn, reduces the connectivity between the sensory and limbic systems and, in that way, deactivates the affected regions [59].

The endocannabinoid system is active in both central and peripheral nervous systems and modulates pain at the spinal, supraspinal, and peripheral levels [60,61]. Cannabinoids may be effective in addressing nociplastic pain [62]. Cannabinoids have been put forth as a potential treatment consideration for fibromyalgia symptoms [63,64]. While there is promising evidence that cannabinoids may indeed be safe and effective for fibromyalgia symptoms, there are limitations with their use, particularly, the most appropriate form to use, dosing, and potential adverse effects with long-term exposure [65]. While the general public is increasingly interested in cannabis as an analgesic alternative, there is evidence of cannabis use disorder and comorbid mental health conditions associated with prolonged exposure [66]. There are no guidelines for their use and there is also a concern about recreational use and abuse.

It should be noted that cannabinoids are relatively contraindicated for those under the age of 21 years and in people with a history of or active substance use disorder, mental health condition, congestive heart failure or cardiovascular disease/risk factors, and in people suffering from palpitations and/or chest pain. Cannabinoids may be associated with mild to severe adverse events, such as dizziness, drowsiness, hypotension, hypoglycemia, disturbed sleep, tachycardia, cardiac palpitations, anxiety, sweating, and psychosis [61].

When used in a balanced way, cannabinoids may rightly be considered for managing fibromyalgia symptoms despite the lack of evidence, particularly in patients suffering from chronic painful symptoms for which there is little other source of relief. When effective, cannabinoids may be opioid-sparing pain relievers.

When considering cannabinoids for a fibromyalgia patient, the clinician must be open and empathetic to the patient while still being frank about potential risks and benefits. Many people regard cannabis products as essentially harmless, which is not the proper attitude for analgesic treatment [65]. Clinicians must also describe appropriate use and dosing. A shared decision-making model is optimal, in which the patient and family members can consider the relative risks, benefits, and possible alternative treatments [67,68]. Note that patients should use cannabinoids only under medical supervision.

Nonpharmacologic Approaches

Exercise, particularly aerobic exercise and resistance training, improves function in fibromyalgia patients and can reduce pain intensity, in addition to the other well-known benefits of exercise such as improved mood, enhanced flexibility, and weight loss. The general recommendation is exercising for at least 20 minutes once a day, two or three times per week. For those who cannot exercise 20 minutes, two 10-minute exercise periods per day may be substituted, but patients should strive for 20 minutes in one day. Strength training at intensities of 40%-80% with 4-20 repetitions per exercise is recommended two to three times weekly [31,69,70].

Cognitive behavioral therapy (CBT) uses a paradigm whereby fibromyalgia is regarded as a biopsychosocial phenomenon in which an overactive emotional system overwhelms a less functional coping system. This so-called salience network means that the body is continuously on a high alert against threats that it cannot manage [71]. CBT can intervene to restore a more holistic balance, and has been found in a meta-analysis to offer ≥50% pain relief and significantly improve negative moods, functional deficits, insomnia, and fatigue [72]. CBT has been shown particularly beneficial for sleep improvement [73], but it requires a commitment of time and effort on the part of the patient and clinical team.

Complementary and alternative medicine offers approaches that may be helpful for those patients interested or open to these treatments. Such treatments include yoga, T’ai Chi, Qi Gong, acupuncture, cupping, traditional Chinese medicine, mindfulness, transcranial stimulation, and others [74-79]. There is a paucity of nutritional guidelines or advice for fibromyalgia patients, although weight control and diets high in plant-based foods and antioxidants hold promise [80-83]. A fibromyalgia diet has been proposed based on the Mediterranean diet; this eating plan can be used to facilitate weight loss and complement other treatment approaches [81]. The EULAR guidance for fibromyalgia treatment recommends exercise, CBT, physical therapies such as hydrotherapy or acupuncture, and meditative movement therapies as nonpharmacologic options [31].

Discussion

Despite our progress and ongoing studies, there remain a couple of unanswered questions about our approach to fibromyalgia.

Who Should Care for Fibromyalgia Patients?

People with fibromyalgia complain about poorly coordinated care and it is known that they can experience diagnostic and treatment delays [27]. Fibromyalgia patients enter the healthcare system with the heavy baggage of having a condition that some healthcare professionals doubt exists or exists in a much milder form than they report. Many fibromyalgia patients feel the healthcare system does not believe them or take them seriously [84]. Affirmative care, supportive clinical teams, and the shared decision-making model for treatment choices may cause fibromyalgia patients to be more satisfied with their healthcare experiences. However, there is no model of care for these patients [67,68]. A multidisciplinary approach that allows for occupational therapy and primary care, and includes specialists in rheumatology or neurology, psychologists or psychiatrics, and nurse consultants may be necessary [2].

What Kinds of Combinations Are Best in Combination Therapy for Fibromyalgia?

Despite limited studies in this important area, it appears that exercise is a foundational component of all combination approaches. To this, educational interventions, CBT, mindfulness exercises, and in some cases pharmacologic therapy with pregabalin and/or an antidepressant can be added [85-87]. While the exact optimal treatment combination has not been determined, there are many elements in the armamentarium and an individualized plan of treatment can be developed. In this connection, it is crucial to bear in mind that fibromyalgia adversely affects sleep, mood, function, and cognition, which should all be recognized in planning treatment [88]. It is also important to recognize that fibromyalgia in some patients may be one component of a much larger system of overlapping pain conditions that can challenge diagnosis and later complicate care.

How Do We Best Care for Fibromyalgia Patients?

The best approach is to assure the patient gets a rapid and accurate diagnosis and can move forward to treatment. It is important to be pragmatic and open with the patient and explain that the diagnosis can be difficult and, once diagnosed, there is no simple treatment for fibromyalgia. A differential diagnosis is complicated by the fact that fibromyalgia mimics the symptoms of other conditions and often occurs in a comorbid relationship with other conditions. During the diagnostic process, clinicians should be professionally empathetic to patients and careful not to dismiss their reports of symptoms, even if they seem unusual or improbable. If fibromyalgia is the appropriate diagnosis, it is imperative that the clinical team work to educate the patient on treatment options, the role of combination therapy, the importance of exercise, medications, and the nature of overlapping symptoms.

## Conclusions

Fibromyalgia can be a devastating diagnosis, often exacerbated by diagnostic delays, clinical skepticism, and a lack of first-line treatment options. Fibromyalgia often exists embedded in a web of overlapping symptoms, confounding diagnosis and treatment. Optimal treatment options seem to involve combination approaches based on a foundation of exercise, weight loss, CBT, and drug therapy. Further studies are needed to better diagnose and treat this perplexing condition that is associated with pain, functional deficits, depressed mood, and disability.

## References

[1] Choy E, Perrot S, Leon T, Kaplan J, Petersel D, Ginovker A, Kramer E (2010). A patient survey of the impact of fibromyalgia and the journey to diagnosis. BMC Health Serv Res.

[2] Sarzi-Puttini P, Giorgi V, Atzeni F (2021). Fibromyalgia position paper. Clin Exp Rheumatol.

[3] Clauw DJ (2014). Fibromyalgia: a clinical review. JAMA.

[4] Coaccioli S, Varrassi G, Sabatini C, Marinangeli F, Giuliani M, Puxeddu A (2008). Fibromyalgia: nosography and therapeutic perspectives. Pain Pract.

[5] Ornsteen AM (1935). Chronic generalized fibromyositis. Ann Surg.

[6] Graham W (1954). Fibrositis and non-articular rheumatism. S Afr J Physiother.

[7] Wolfe F, Walitt B (2013). Culture, science and the changing nature of fibromyalgia. Nat Rev Rheumatol.

[8] Perrot S (2012). If fibromyalgia did not exist, we should have invented it. A short history of a controversial syndrome. Reumatismo.

[9] Sluka KA, Clauw DJ (2016). Neurobiology of fibromyalgia and chronic widespread pain. Neuroscience.

[10] Albrecht DS, Forsberg A, Sandström A (2019). Brain glial activation in fibromyalgia - a multi-site positron emission tomography investigation. Brain Behav Immun.

[11] Schmidt-Wilcke T, Clauw DJ (2011). Fibromyalgia: from pathophysiology to therapy. Nat Rev Rheumatol.

[12] Lichtenstein A, Tiosano S, Amital H (2018). The complexities of fibromyalgia and its comorbidities. Curr Opin Rheumatol.

[13] Fitzcharles MA, Perrot S, Häuser W (2018). Comorbid fibromyalgia: a qualitative review of prevalence and importance. Eur J Pain.

[14] Yunus MB (2008). Central sensitivity syndromes: a new paradigm and group nosology for fibromyalgia and overlapping conditions, and the related issue of disease versus illness. Semin Arthritis Rheum.

[15] Riedl A, Schmidtmann M, Stengel A, Goebel M, Wisser AS, Klapp BF, Mönnikes H (2008). Somatic comorbidities of irritable bowel syndrome: a systematic analysis. J Psychosom Res.

[16] Johnson CM, Makai GE (2018). Fibromyalgia and irritable bowel syndrome in female pelvic pain. Semin Reprod Med.

[17] Birder LA (2019). Pathophysiology of interstitial cystitis. Int J Urol.

[18] Nickel JC, Tripp DA, Pontari M (2010). Interstitial cystitis/painful bladder syndrome and associated medical conditions with an emphasis on irritable bowel syndrome, fibromyalgia and chronic fatigue syndrome. J Urol.

[19] Okifuji A, Donaldson GW, Barck L, Fine PG (2010). Relationship between fibromyalgia and obesity in pain, function, mood, and sleep. J Pain.

[20] Patucchi E, Fatati G, Puxeddu A, Coaccioli S (2003). Prevalence of fibromyalgia in diabetes mellitus and obesity. (Article in Italian). Recenti Progr Med.

[21] Zhao SS, Duffield SJ, Goodson NJ (2019). The prevalence and impact of comorbid fibromyalgia in inflammatory arthritis. Best Pract Res Clin Rheumatol.

[22] Whealy M, Nanda S, Vincent A, Mandrekar J, Cutrer FM (2018). Fibromyalgia in migraine: a retrospective cohort study. J Headache Pain.

[23] Küçükşen S, Genç E, Yılmaz H (2013). The prevalence of fibromyalgia and its relation with headache characteristics in episodic migraine. Clin Rheumatol.

[24] de Tommaso M, Federici A, Serpino C (2011). Clinical features of headache patients with fibromyalgia comorbidity. J Headache Pain.

[25] Penn IW, Chuang E, Chuang TY, Lin CL, Kao CH (2019). Bidirectional association between migraine and fibromyalgia: retrospective cohort analyses of two populations. BMJ Open.

[26] Häuser W, Sarzi-Puttini P, Fitzcharles MA (2019). Fibromyalgia syndrome: under-, over- and misdiagnosis. Clin Exp Rheumatol.

[27] Sarzi-Puttini P, Giorgi V, Atzeni F (2021). Diagnostic and therapeutic care pathway for fibromyalgia. Clin Exp Rheumatol.

[28] Häuser W, Fitzcharles MA (2018). Facts and myths pertaining to fibromyalgia. Dialogues Clin Neurosci.

[29] Arnold LM, Clauw DJ, McCarberg BH (2011). Improving the recognition and diagnosis of fibromyalgia. Mayo Clin Proc.

[30] Tirelli U, Cirrito C, Pavanello M, Piasentin C, Lleshi A, Taibi R (2019). Ozone therapy in 65 patients with fibromyalgia: an effective therapy. Eur Rev Med Pharmacol Sci.

[31] Macfarlane GJ, Kronisch C, Atzeni F (2017). EULAR recommendations for management of fibromyalgia. Ann Rheum Dis.

[32] Carville SF, Arendt-Nielsen L, Bliddal H (2008). EULAR evidence-based recommendations for the management of fibromyalgia syndrome. Ann Rheum Dis.

[33] Siler AC, Gardner H, Yanit K, Cushman T, McDonagh M (2011). Systematic review of the comparative effectiveness of antiepileptic drugs for fibromyalgia. J Pain.

[34] Cooper TE, Derry S, Wiffen PJ, Moore RA (2017). Gabapentin for fibromyalgia pain in adults. Cochrane Database Syst Rev.

[35] Tzellos TG, Toulis KA, Goulis DG, Papazisis G, Zampeli VA, Vakfari A, Kouvelas D (2010). Gabapentin and pregabalin in the treatment of fibromyalgia: a systematic review and a meta-analysis. J Clin Pharm Ther.

[36] McClure EW, Daniels RN (2021). Classics in chemical neuroscience: amitriptyline. ACS Chem Neurosci.

[37] Perrot S, Russell IJ (2014). More ubiquitous effects from non-pharmacologic than from pharmacologic treatments for fibromyalgia syndrome: a meta-analysis examining six core symptoms. Eur J Pain.

[38] Rico-Villademoros F, Slim M, Calandre EP (2015). Amitriptyline for the treatment of fibromyalgia: a comprehensive review. Expert Rev Neurother.

[39] Lunn MP, Hughes RA, Wiffen PJ (2014). Duloxetine for treating painful neuropathy, chronic pain or fibromyalgia. Cochrane Database Syst Rev.

[40] Elahi N, Ellahi A (2023). Duloxetine: an effective treatment for fibromyalgia syndrome?. IJS Global Health.

[41] Gupta H, Girma B, Jenkins JS, Kaufman SE, Lee CA, Kaye AD (2021). Milnacipran for the treatment of fibromyalgia. Health Psychol Res.

[42] Derry S, Phillips T, Moore RA, Wiffen PJ (2015). Milnacipran for neuropathic pain in adults. Cochrane Database Syst Rev.

[43] Cording M, Derry S, Phillips T, Moore RA, Wiffen PJ (2015). Milnacipran for pain in fibromyalgia in adults. Cochrane Database Syst Rev.

[44] Alberti FF, Becker MW, Blatt CR, Ziegelmann PK, da Silva Dal Pizzol T, Pilger D (2022). Comparative efficacy of amitriptyline, duloxetine and pregabalin for treating fibromyalgia in adults: an overview with network meta-analysis. Clin Rheumatol.

[45] Baraniuk JN, Whalen G, Cunningham J, Clauw DJ (2004). Cerebrospinal fluid levels of opioid peptides in fibromyalgia and chronic low back pain. BMC Musculoskelet Disord.

[46] Goldenberg DL, Clauw DJ, Palmer RE, Clair AG (2016). Opioid use in fibromyalgia: a cautionary tale. Mayo Clin Proc.

[47] Häuser W, Thieme K, Turk DC (2010). Guidelines on the management of fibromyalgia syndrome - a systematic review. Eur J Pain.

[48] Dydyk AM, Jain NK, Gupta M (2023). Opioid use disorder. StatPearls [Internet].

[49] Bruce BK, Allman ME, Rivera FA (2021). Opioid use in fibromyalgia continues despite guidelines that do not support its efficacy or risk. J Clin Rheumatol.

[50] Bennett RM, Kamin M, Karim R, Rosenthal N (2003). Tramadol and acetaminophen combination tablets in the treatment of fibromyalgia pain: a double-blind, randomized, placebo-controlled study. Am J Med.

[51] Younger J, Noor N, McCue R, Mackey S (2013). Low-dose naltrexone for the treatment of fibromyalgia: findings of a small, randomized, double-blind, placebo-controlled, counterbalanced, crossover trial assessing daily pain levels. Arthritis Rheum.

[52] Parkitny L, Younger J (2017). Reduced pro-inflammatory cytokines after eight weeks of low-dose naltrexone for fibromyalgia. Biomedicines.

[53] Yang J, Shin KM, Do A (2023). The safety and efficacy of low-dose naltrexone in patients with fibromyalgia: a systematic review. J Pain Res.

[54] Yunus MB, Masi AT, Aldag JC (1989). Short term effects of ibuprofen in primary fibromyalgia syndrome: a double blind, placebo controlled trial. J Rheumatol.

[55] Choy E, Marshall D, Gabriel ZL, Mitchell SA, Gylee E, Dakin HA (2011). A systematic review and mixed treatment comparison of the efficacy of pharmacological treatments for fibromyalgia. Semin Arthritis Rheum.

[56] Arrighi E, Ruiz de Castilla EM, Peres F (2022). Scoping health literacy in Latin America. Glob Health Promot.

[57] Ablin JN, Elkayam O, Fitzcharles MA (2016). Attitudes of Israeli rheumatologists to the use of medical cannabis as therapy for rheumatic disorders. Rambam Maimonides Med J.

[58] Clayton N, Marshall FH, Bountra C, O'Shaughnessy CT (2002). CB1 and CB2 cannabinoid receptors are implicated in inflammatory pain. Pain.

[59] Walter C, Oertel BG, Felden L (2016). Brain mapping-based model of Δ(9)-tetrahydrocannabinol effects on connectivity in the pain matrix. Neuropsychopharmacology.

[60] Sarzi-Puttini P, Batticciotto A, Atzeni F (2019). Medical cannabis and cannabinoids in rheumatology: where are we now?. Expert Rev Clin Immunol.

[61] Sarzi-Puttini P, Ablin J, Trabelsi A, Fitzcharles MA, Marotto D, Häuser W (2019). Cannabinoids in the treatment of rheumatic diseases: pros and cons. Autoimmun Rev.

[62] Fitzcharles MA, Petzke F, Tölle TR, Häuser W (2021). Cannabis-based medicines and medical cannabis in the treatment of nociplastic pain. Drugs.

[63] Bell AD, MacCallum C, Margolese S (2023). Clinical practice guidelines for cannabis and cannabinoid-based medicines in the management of chronic pain and co-occurring conditions. Cannabis Cannabinoid Res.

[64] Bourke SL, Schlag AK, O'Sullivan SE, Nutt DJ, Finn DP (2022). Cannabinoids and the endocannabinoid system in fibromyalgia: a review of preclinical and clinical research. Pharmacol Ther.

[65] Khurshid H, Qureshi IA, Jahan N, Went TR, Sultan W, Sapkota A, Alfonso M (2021). A systematic review of fibromyalgia and recent advancements in treatment: is medicinal cannabis a new hope?. Cureus.

[66] Hasin D, Walsh C (2021). Cannabis use, cannabis use disorder, and comorbid psychiatric illness: a narrative review. J Clin Med.

[67] Elwyn G, Frosch D, Thomson R (2012). Shared decision making: a model for clinical practice. J Gen Intern Med.

[68] Doebl S, Macfarlane GJ, Hollick RJ (2020). "No one wants to look after the fibro patient". Understanding models, and patient perspectives, of care for fibromyalgia: reviews of current evidence. Pain.

[69] Pinto AM, Geenen R, Castilho P, da Silva JA (2020). Progress towards improved non-pharmacological management of fibromyalgia. Joint Bone Spine.

[70] da Silva JM, de Barros BS, Almeida GJ, O'Neil J, Imoto AM (2022). Dosage of resistance exercises in fibromyalgia: evidence synthesis for a systematic literature review up-date and meta-analysis. Rheumatol Int.

[71] Pinto AM, Geenen R, Wager TD (2023). Emotion regulation and the salience network: a hypothetical integrative model of fibromyalgia. Nat Rev Rheumatol.

[72] Bernardy K, Klose P, Welsch P, Häuser W (2018). Efficacy, acceptability and safety of cognitive behavioural therapies in fibromyalgia syndrome - a systematic review and meta-analysis of randomized controlled trials. Eur J Pain.

[73] Prados G, Miró E, Martínez MP, Sánchez AI, Lami MJ, Cáliz R (2020). Combined cognitive-behavioral therapy for fibromyalgia: effects on polysomnographic parameters and perceived sleep quality. Int J Clin Health Psychol.

[74] Cao HJ, Zhang YJ, Zhou L (2020). Partially randomized patient preference trial: comparative evaluation of fibromyalgia between acupuncture and cupping therapy (PRPP-FACT). Complement Ther Clin Pract.

[75] Cao H, Hu H, Colagiuri B, Liu J (2011). Medicinal cupping therapy in 30 patients with fibromyalgia: a case series observation. Forsch Komplementmed.

[76] Cao H, Liu J, Lewith GT (2010). Traditional Chinese Medicine for treatment of fibromyalgia: a systematic review of randomized controlled trials. J Altern Complement Med.

[77] Fischer-White TG, Anderson JG, Taylor AG (2016). An integrated methodology to assess compliance with Delphi survey key components of yoga interventions for musculoskeletal conditions as applied in a systematic review of fibromyalgia studies. Explore (NY).

[78] Berger AA, Liu Y, Nguyen J (2021). Efficacy of acupuncture in the treatment of fibromyalgia. Orthop Rev (Pavia).

[79] Lloyd DM, Wittkopf PG, Arendsen LJ, Jones AK (2020). Is transcranial direct current stimulation (tDCS) effective for the treatment of pain in fibromyalgia? A systematic review and meta-analysis. J Pain.

[80] Lowry E, Marley J, McVeigh JG, McSorley E, Allsopp P, Kerr D (2020). Dietary interventions in the management of fibromyalgia: a systematic review and best-evidence synthesis. Nutrients.

[81] Pagliai G, Giangrandi I, Dinu M, Sofi F, Colombini B (2020). Nutritional interventions in the management of fibromyalgia syndrome. Nutrients.

[82] Silva AR, Bernardo A, de Mesquita MF, Vaz-Patto J, Moreira P, Silva ML, Padrão P (2022). An anti-inflammatory and low fermentable oligo, di, and monosaccharides and polyols diet improved patient reported outcomes in fibromyalgia: a randomized controlled trial. Front Nutr.

[83] Maddox EK, Massoni SC, Hoffart CM, Takata Y (2023). Dietary effects on pain symptoms in patients with fibromyalgia syndrome: systematic review and future directions. Nutrients.

[84] Briones-Vozmediano E, Vives-Cases C, Ronda-Pérez E, Gil-González D (2013). Patients' and professionals' views on managing fibromyalgia. Pain Res Manag.

[85] Serrat M, Sanabria-Mazo JP, Almirall M (2021). Effectiveness of a multicomponent treatment based on pain neuroscience education, therapeutic exercise, cognitive behavioral therapy, and mindfulness in patients with fibromyalgia (FIBROWALK study): a randomized controlled trial. Phys Ther.

[86] Nüesch E, Häuser W, Bernardy K, Barth J, Jüni P (2013). Comparative efficacy of pharmacological and non-pharmacological interventions in fibromyalgia syndrome: network meta-analysis. Ann Rheum Dis.

[87] Ollevier A, Vanneuville I, Carron P, Baetens T, Goderis T, Gabriel L, Van de Velde D (2020). A 12-week multicomponent therapy in fibromyalgia improves health but not in concomitant moderate depression, an exploratory pilot study. Disabil Rehabil.

[88] Mease PJ, Clauw DJ, Christensen R (2011). Toward development of a fibromyalgia responder index and disease activity score: OMERACT module update. J Rheumatol.

